# Insights into the epigenetics of chronic rhinosinusitis with and without nasal polyps: a systematic review

**DOI:** 10.3389/falgy.2023.1165271

**Published:** 2023-05-22

**Authors:** Tripti Brar, Lisa Marks, Devyani Lal

**Affiliations:** ^1^ Division of Rhinology, Department of Otolaryngology, Mayo Clinic in Arizona, Phoenix, AZ, United States; ^2^ Division of Education, Department of Library Services, Mayo Clinic, Phoenix, AZ, United States

**Keywords:** epigenetics, CRSwNP, sinusitis, nasal polyps, chronic rhinosinusitis, DNA methylation, miRNA, non-coding RNA

## Abstract

**Background:**

Epigenetics facilitates insights on the impact of host environment on the genesis of chronic rhinosinusitis (CRS) through modulations of host gene expression and activity. Epigenetic mechanisms such as DNA methylation cause reversible but heritable changes in gene expression over generations of progeny, without altering the DNA base-pair sequences. These studies offer a critical understanding of the environment-induced changes that result in host predisposition to disease and may help in developing novel biomarkers and therapeutics. The goal of this systematic review is to summarize the current evidence on epigenetics of CRS with a focus on chronic rhinosinusitis with nasal polyps (CRSwNP) and highlight gaps that merit further research.

**Methods:**

A systematic review of the English language literature was performed to identify investigations related to epigenetic studies in subjects with CRS.

**Results:**

The review identified 65 studies. These have focused on DNA methylation and non-coding RNAs, with only a few on histone deacetylation, alternative polyadenylation, and chromatin accessibility. Studies include those investigating *in vivo* and *in vitro* changes or both. Studies also include animal models of CRS. Almost all have been conducted in Asia. The genome-wide studies of DNA methylation found differences in global methylation between CRSwNP and controls, while others specifically found significant differences in methylation of the CpG sites of the thymic stromal lymphopoietin (*TSLP*), *IL-8*, and *PLAT*. In addition, DNA methyltransferase inhibitors and histone deacetylase inhibitors were studied as potential therapeutic agents. Majority of the studies investigating non-coding RNAs focused on micro-RNAs (miRNA) and found differences in global expression of miRNA levels. These studies also revealed some previously known as well as novel targets and pathways such as tumor necrosis factor alpha, TGF beta-1, IL-10, *EGR2*, aryl hydrocarbon receptor, PI3K/AKT pathway, mucin secretion, and vascular permeability. Overall, the studies have found a dysregulation in pathways/genes involving inflammation, immune regulation, tissue remodeling, structural proteins, mucin secretion, arachidonic acid metabolism, and transcription.

**Conclusions:**

Epigenetic studies in CRS subjects suggest that there is likely a major impact of the environment. However, these are association studies and do not directly imply pathogenesis. Longitudinal studies in geographically and racially diverse population cohorts are necessary to quantify genetic vs. environmental risks for CRSwNP and CRS without nasal polyps and assess heritability risk, as well as develop novel biomarkers and therapeutic agents.

## Introduction

**Figure 1:**
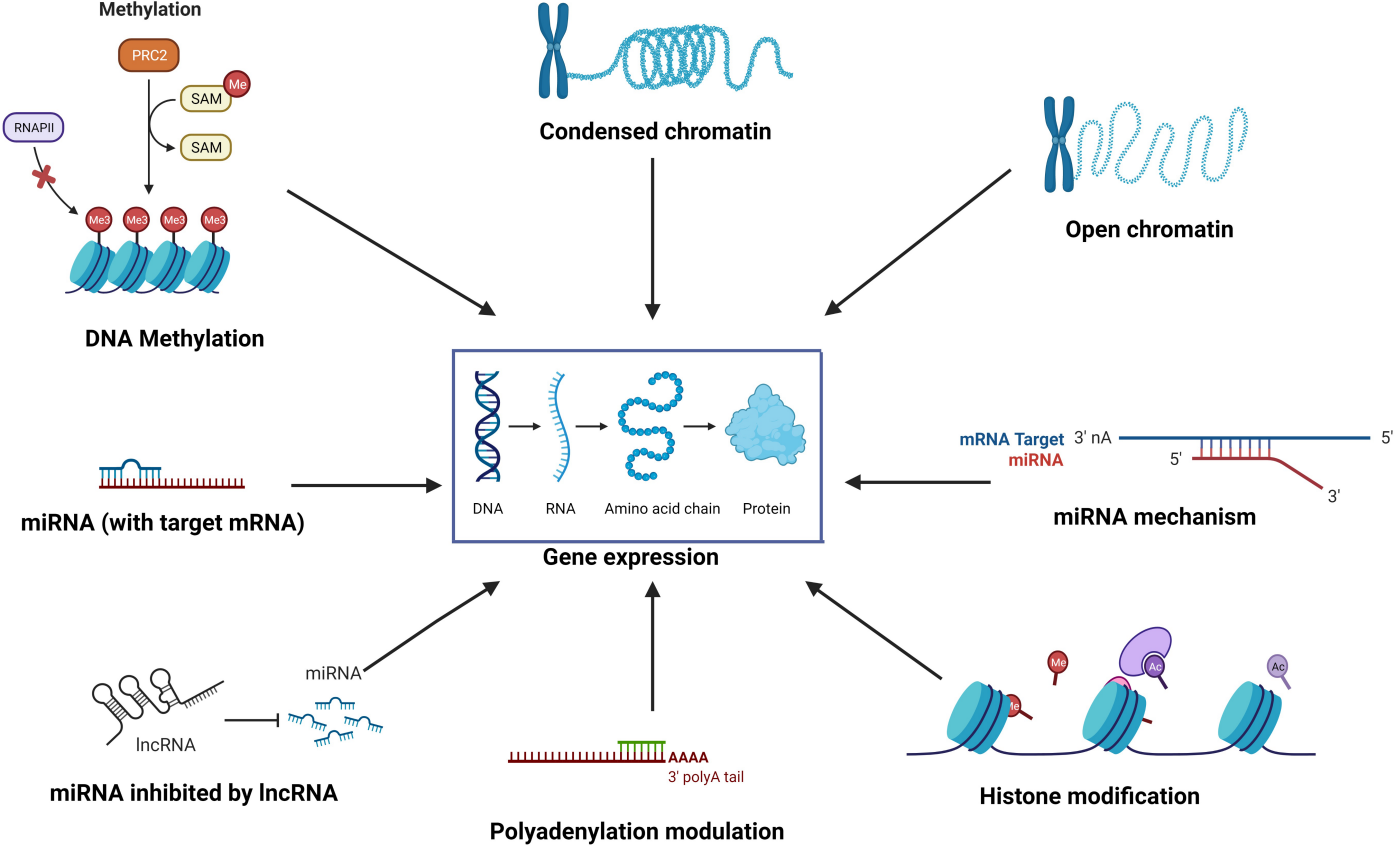
The environment affects the “epigenome” which in turn affects gene expression through various epigenetic mechanisms such as DNA methylation, histone modification, role of non-coding RNAs such as miRNA and lncRNA, alternative polyadenylation, and chromatin accessibility.

Epigenetics is the study of reversible molecular mechanisms that modify gene activity and expression without altering the DNA base-pair nucleotide structure. These epigenetic changes are a consequence of the impact of the host environment. The commonly involved mechanisms through which epigenetic modulations are deployed include DNA methylation, histone modifications, non-coding RNAs, and alternative polyadenylation (APA). DNA methylation decreases gene expression by blocking specific sites on DNA, which prevents the binding of transcription factors on the DNA (1). Thus, hypermethylation may repress gene transcription, and hypomethylation potentially increases gene transcription. Histone modifications also control transcription by regulating the winding and unwinding of DNA. Tightly coiled DNA around histones is unable to undergo transcription. Chemical modifications (acetylation and deacetylation) in histones can alter the amount of DNA available for transcription (2). In general, histone acetylation causes transcriptional activation, and deacetylation causes transcriptional repression (3). Non-coding RNAs such as micro-RNAs (miRNAs) and long non-coding RNA (lncRNA) may control post-transcription protein synthesis, in addition to transcription. miRNAs can bind to target mRNAs leading to their degradation. Epi-miRNAs can repress key enzymes that drive epigenetic remodeling and also bind to complementary sequences in gene promoters, recruiting specific protein complexes that modulate chromatin structure and gene expression. miRNAs themselves can also be epigenetically regulated by DNA methylation and/or specific histone modifications (4). In turn, non-coding RNAs also have regulatory epigenetic roles. Non-coding RNAs such as lncRNAs and circular RNAs (circRNAs) can affect the methylation pattern of downstream genes (5), and short interfering RNA (siRNAs) and miRNAs can lead to transcriptional gene silencing through DNA methylation and histone modifications (6). APA at the 3′ untranslated region (UTR) affects mRNA stability and protein translation (7). Thus, the environment (microbes, allergens, pollutants, smoke, etc.) can induce changes in the “epigenome” through various mechanisms, ultimately affecting gene expression without disturbing the “genome”. Figure 1 illustrates some of the common epigenetic mechanisms that have been investigated in chronic rhinosinusitis (CRS).

While epigenetic changes occur as a result of the host–environment interaction, but they can be passed on to progeny for a few generations (8). However, because these changes are pharmacogenetically reversible (9), there exists a huge potential in developing biomarkers and novel treatments and optimizing personalized therapeutics. Currently, seven epigenetic agents are FDA-approved for treatment of certain cancers, while research on several other drugs is underway for oncologic and non-oncologic indications (10).

CRS is a common condition requiring surgical management in the United States and worldwide, with many subtypes being particularly recalcitrant to standard of care treatment modalities (11, 12). The disease is heterogenous in both clinical features as well as underlying etiopathogenic factors. Factors that may attribute to disease pathophysiology include altered host responses at epithelial surfaces, which may lead to changes in epithelial barrier integrity and permeability and altered innate and adaptive immune responses (13). The role of the epithelium cannot be emphasized enough, as it is also the interface for the host–environment interaction.

CRS with nasal polyps (CRSwNP) is a subtype that has been shown to be associated with familial clustering (14, 15). Whether this clustering reflects genetic susceptibility (16) or a shared and common environment is yet to be fully deciphered as familial clustering also affects spouses (14). Thus, understanding the role of both genetic susceptibility and the environment is important in CRS. The study of epigenetics is thus valuable in CRS as it can help elucidate genetic factors that are driven through environmental changes rather than changes in the actual gene structure (17). Recent reviews on the role of epigenetics in CRS have emphasized the immense potential of these studies in laying the foundation to the development of novel therapeutics in CRS (17, 18). Thus, the identification of these reversible mechanisms that can alter gene expression can lead to a paradigm shift in treatment approaches in the future.

The goal of our systematic review was to search the literature for all studies that have investigated the role of epigenetic mechanisms in CRS and CRSwNP, since a better understanding of these may assist with quantifying the disease risk and prognostication, as well as developing biomarkers and personalized treatment for the management of this condition.

## Methods

A review of the English language literature was performed on PubMed to identify all original studies that investigated epigenetic mechanisms in CRS from inception to September 2022. References of these articles were also further reviewed to include additional original reports on epigenetics in CRS. For this project, a medical librarian searched the following databases: Ovid MEDLINE, Ovid EMBASE, Scopus, and Web of Science. All searches were conducted in January 2023, with no date limitations but limited to English language. The search was a combination of Medical Subject Headings (MeSH) and keywords. The MeSH terms used included the following: Epigenesis, Genetic; DNA Methylation; MicroRNAs; Histones; RNA, Untranslated; Polyadenylation; Sinusitis; and Nasal Polyps. The keywords used included the following: epigenetic*; methylation; micro-RNA*; MiR; histone; non coding rna; non-coding rna; noncoding rna; alternative polyadenylation; lncrna; sinusitis; nasal polyp*, and chronic rhinosinusitis. The epigenetic terms were combined using the Boolean operator “OR”, and the sinusitis terms were also combined using the Boolean operator of “OR”. The epigenetic terms and the sinusitis terms were then combined using the Boolean operator “AND” resulting in 790 references. Duplicate references were then removed leaving a total of 420 references for review to meet the inclusion or exclusion criteria. (* indicates truncation of word or phrase).

After screening the titles and abstracts, we retrieved published full-text articles and extracted data. Two independent reviewers reviewed the articles for inclusion, and disagreements were resolved by consensus.

## Results

**Figure 2:**
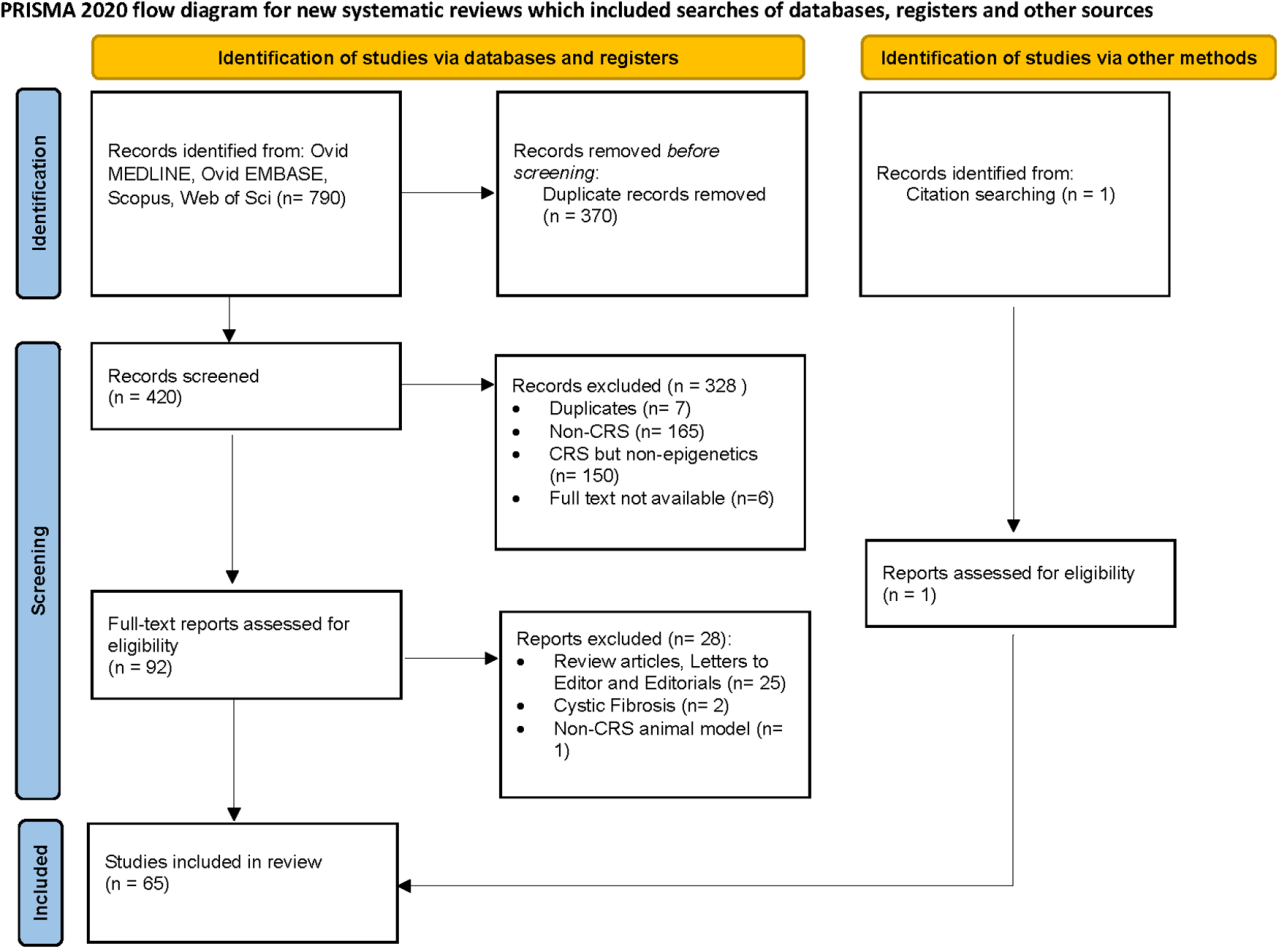
Preferred reporting items for systematic reviews and meta-analyses (PRISMA) diagram.

**Figure 3:**
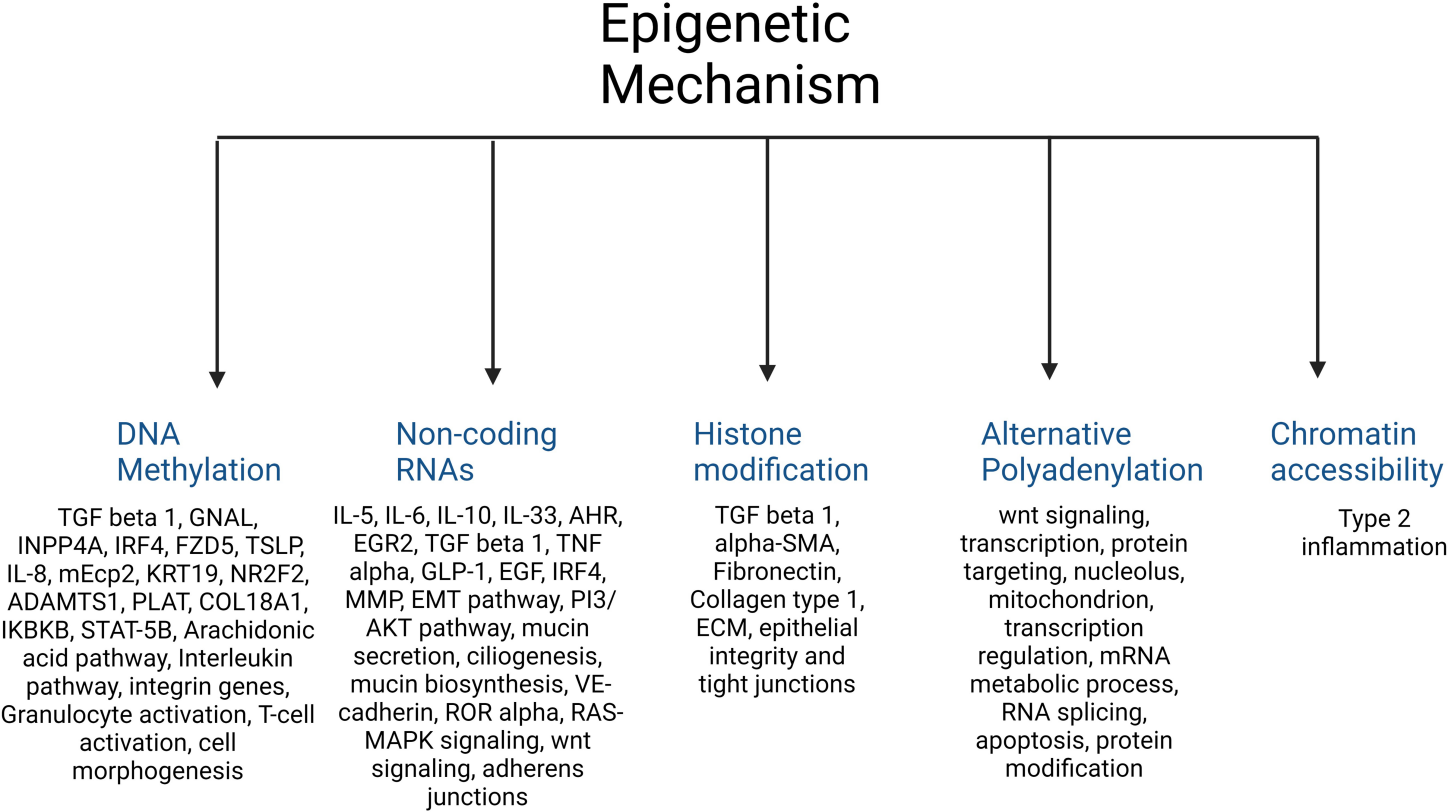
Illustration of the major epigenetic mechanisms investigated in CRS and a summary of the main genes, proteins, or pathways that were identified in each.

A total of 420 studies were reviewed. After excluding non-CRS studies, non-epigenetic studies, studies without full texts, duplicates, review articles, editorials, and cystic fibrosis, we identified 65 studies that investigated the role of epigenetics in CRS. Please refer to PRISMA diagram illustrated in Figure 2. A majority of studies investigated the role of non-coding RNAs (45 studies) followed by DNA methylation (13 studies). Four studies investigated histone modifications, two studies were on APA, and one study on chromatin accessibility. Most studies focused on CRSwNP, while very few included CRS agnostic of polyp status or CRS without nasal polyps (CRSsNP). Most studies were reported on populations from China (44) followed by Korea (12), Japan (1), Spain (2), Belgium (1), Brazil (2), Poland (1), and the United States (2). Details of the studies are presented below, categorized by the epigenetic mechanism investigated and listed in Tables 1–4 and Supplementary Table S1. Figure 3 illustrates the epigenetic mechanism investigated in CRS and summarizes the genes, proteins, or pathways that were identified or studied.

**Table 1:** DNA methylation studies in chronic rhinosinusitis.

No.	Author/year	Subjects	Country of study	Tissue analyzed	*In vivo*/*in vitro*/both	Validation	Broad results	Specific genes/biological pathway identified or investigated
1.	Park et al. (19)	CRSsNP-UP: 10; CRSwNP-UP: 10; CRSwNP-NP: 13; Controls: 4	Korea	UP in CRSwNP, CRSsNP; NP in CRSwNP; “nasal mucosa tissue” in controls Purified HNECs extracted for *ex vivo* air–liquid interface cell cultures.	Both	RNA and protein levels validated by RT-PCR and Western blotting.	Global DNA methylation and DNMT activity were increased in CRSwNP and CRSsNP vs. controls. TGF beta-1 dose-dependently induced DNMT expression and global DNA methylation. 5-azacytidine inhibited TGF beta-1-induced DNMT in primary HNECs and air–liquid interface cultures. Inhibition of DNMT suppressed the EMT process and therefore may be potentially a CRS therapeutic strategy.	TGF beta-1 (regulates cell proliferation, differentiation and growth, immune function, can modulate expression and activation of other growth factors including interferon gamma and tumor necrosis factor alpha).
2.	Tan et al. (20)	eCRSwNP: 19; neCRSwNP: 19; Controls: 19	China	NP in CRSwNP; MTM in controls.	*In vivo*	Validated expression of selected DhMGs on a smaller sub-group, using RT-PCR and tissue IHC.	Between eCRSwNP and neCRSwNP, a total of 26 DhMGs were identified. Compared with controls, 169 DhMGs were identified in eCRSwNP. GNAL, INPP4A, and IRF4 expression levels were significantly different between eCRSwNP and the other two groups Only one DhMG, *DACH2*, was detected between neCRSwNP and the control.	GNAL (encodes a stimulatory G protein alpha subunit that mediates odorant signaling in the olfactory epithelium); INPP4A (encodes an enzyme in the inositol metabolism pathway); and IRF4 (interferon regulatory factor and regulation of interferon-inducible genes).
3.	Kim et al. (21)	eCRSwNP: 57; neCRSwNP: 72; Controls: 18	Korea	NPs from CRSwNP; “normal nasal tissue” from controls	*In vivo*	—	44 DMP-enriched genes in eCRSwNP including IL13, Ras and Rab interactor 3 (RIN3), CEBPE, IL3, and ACOT7. For each gene, the average methylation levels were lower in cases than in controls.	Pathways: granulocyte activation, T-cell activation, and leukocyte migration, leukocyte migration and regulation of cell morphogenesis were significantly enriched on tissue eosinophilia analysis.
4.	Kim et al. (22)	eCRSwNP: 7; neCRSwNP: 8; Controls: 8	Korea	UP in CRSwNP and CRSsNP; NP in CRSwNP; UP in controls, HNECs also isolated and cell cultures prepared.	Both	Validated expression of some genes using RT-PCR and proteins using Western blotting. The mRNA level of FZD5 was higher in the eCRSwNP than neCRSwNP group.	397 and 387 genes were differentially hypermethylated in the eCRSwNP and neCRSwNP groups respectively, compared with controls. 399 and 208 genes were hypomethylated in the eCRSwNP and neCRSwNP respectively, compared with controls. Between eCRSwNP and neCRSwNP, 4 genes exhibited hypermethylation in eCRSwNP and 19 genes exhibited hypomethylation. FZD5 significantly hypomethylated in eCRSwNP compared with neCRSwNP and controls.	FZD5 gene (receptor for Wnt5A, and its complex with Wnt5A contributes to activating inflammation and tissue modification).
5.	Li et al. (23)	CRSwNP: 48; CRSsNP: 28; Controls: 21	China	NP from CRSwNP, ETM from CRSsNP, ITM from controls. Purified HNECs extracted from these samples	*In vivo*	—	17 CpG units were analyzed in the *TSLP* gene, two CpG units had increased methylation ratios in the CRSwNP patients compared with the CRSsNP and control subjects.	*TSLP* gene (cytokine that promotes Th2 response).
6.	Li et al. (24)	CRSwNP: 187; CRSsNP: 89; Controls: 57	China	NP from CRSwNP, ETM from CRSsNP, ITM from controls. Purified HNECs extracted for *ex vivo* air–liquid interface cell cultures.	Both	Correlated with mRNA levels. Also, *ex vivo* study demonstrated methylation changes that corresponded with IL-8 levels in stimulated (IL-1beta and IL-17) and unstimulated cell cultures.	Two independent cohorts were studied. DNA methylation of CpG sites 1, 2, and 3 in IL-8 proximal promoter was significantly decreased in CRSwNP as compared with CRSsNP. IL-8 levels by ELISA analysis showed significantly higher levels in CRSwNP as compared with CRSsNP and controls. A significantly negative correlation was seen between IL-8 levels and percentage of methylation of all 3 CpG sites.	IL-8 gene (proinflammatory cytokine, chemotactic factor, potent angiogenic factor).
7.	Shin et al. (25)	CRSwNP: 7; Controls: 5	Korea	NP in CRSwNP; IT in Controls; TGFb1-induced NPDFs	*In vitro*	—	Expression levels of methyl-CpG-binding protein 2 (MeCP2), a transcription regulator, were higher in NP vs. control IT. It was suppressed by 5-azacytidine, which had an inhibitory effect on TGFb1 NPDFs and ECM production.	MeCP2 (can bind to methylated DNA, transcription regulation and gene expression).
8.	Kim et al. (26)	CRSwNP: 7; CRSsNP: 7; Controls: 4	Korea	NP from CRSwNP, ITM from all three groups used as controls.	*In vivo*	KRT19 and NR2F2 mRNA were significantly increased in NP samples from all three compared with UP control from CRSsNP and CRSwNP. ADAMTS1 was significantly increased in two of the three patients. ZNF222 levels did not differ significantly.	Promoter regions of 10 and 30 genes were hypermethylated and hypomethylated, respectively, in CRSwNP samples compared with controls. KRT19, NR2F2, and ADAMTS1 were hypomethylated in CRSwNP.	KRT19 (keratin 19: structural integrity); NR2F2 (transcription factor); ADAMTS1 (anti-angiogenic activity).
9.	Kidoguchi et al. (27)	CRSwNP: 20; Control tissue from same patients	Japan	NP, ITM used as control from CRS	*In vivo*	Correlated with mRNA expression	Methylation levels at three CpG sites in the proximal *PLAT* promoter regions were significantly higher, and *PLAT* gene expression was significantly lower in NP compared with ITM, possibly leading to excessive fibrin deposition by an aberrant coagulation cascade.	*PLAT* gene (serine protease that converts the proenzyme plasminogen to plasmin, a fibrinolytic enzyme; participates in coagulation, cell migration, and tissue remodeling).
10.	Zheng et al. (28)	CRSwNP: 32; Controls: 18	China	NP in CRS, ITM in controls	*In vivo*	COL181 methylation changes verified by MSP and bisulfite sequencing. But did not correlate with gene expression and IHC.	198 genes had a methylated signal in the promoter region in patients vs. controls. COL181 promoter was hypermethylated in patients vs. controls.	COL18A1 (collagen type VIII, an ECM protein).
11.	Pérez-Novo et al. (29)	CRSwNP: 3 No Controls	Belgium	NP cultured with SEB	*In vitro*	—	43 genes exhibited changes in the methylation state after 24-h culture with SEB. Of these, 33 genes were hypermethylated while 10 showed hypomethylation.	IKBKB (activator of NF-kappa-B) and STAT-5B (member of the STAT family of transcription factors). Both play a crucial role in T-cell maturation/activation and immune response.
12.	Seiberling et al. (30)	CRSwNP: 14; Controls: 3	United States	NP in CRS; posterior ethmoid in controls.	*In vivo*	—	CRSwNP specimens demonstrate elevated levels of modified cytosine, 5-bromocytosine (5BrC) and 5 chlorocytosine (CIC) compared with normal controls. These may result in the aberrant methylation of cytosine during DNA replication.	
13.	Cheong et al. (31)	CRSwNP with AIA: 5; CRSwNP with ATA: 4	Korea	NP and peripheral blood	*In vivo*	—	Methylation levels in the four sample groups were similar; however DNA methylation profiles differed between ATA and AIA groups. IL5RA, IL20RA, IL22RA, PTGES hypermethylated in AIA and IL2RA, IL10 and CD28, PGDS, ALOX5AP, LTB42 hypomethylated.	Arachidonic acid pathway; interleukin pathway; integrin genes.

AIA, aspirin-intolerant asthma; ATA, aspirin-tolerant asthma; CRS, chronic rhinosinusitis; CpG, cytosine followed by guanine; CRSsNP, chronic rhinosinusitis without nasal polyps; CRSwNP, chronic rhinosinusitis with nasal polyps; DhMG, differentially hydroxymethylated genes; DMP, differentially methylated probes; DNMT, DNA methyltransferase; ECM, extracellular matrix; EMT, epithelial–mesenchymal transition; eCRSwNP, eosinophilic chronic rhinosinusitis with nasal polyps; ETM, ethmoid mucosa; HNEC, human nasal epithelial cells; IHC, immunohistochemistry; ITM, inferior turbinate mucosa; MTM, middle turbinate mucosa; neCRSwNP, non-eosinophilic chronic rhinosinusitis with nasal polyps; NP, nasal polyps; NPDFs, nasal polyp-derived fibroblasts; RT-PCR, reverse transcription polymerase chain reaction; SEB, staphylococcus enterotoxin B; UP, uncinate process; TSLP, thymic stromal lymphopoietin.

**Table 2:** Studies evaluating the role of histone modifications (deacetylation) in chronic rhinosinusitis.

No.	Author/year	Subjects	Country of study	Tissue analyzed	*In vivo*/*in vitro*/both	Validation	Broad results	Specific genes/biological pathway identified or investigated
1	Cho et al. (32)	CRSwNP: 7; Controls: ?	Korea	NP in CRSwNP, ITM in controls (only for comparing mRNA expression of HDAC2, alpha-SMA and TGF beta-1) NPDFs isolated.	*In vitro*	Correlated findings with mRNA and protein levels.	HDAC inhibition using TSA blocked proliferation in TGF beta-1-induced NPDFs.	TGF beta-1, alpha-SMA
2	Cho et al. (33)	CRSwNP: 18	Korea	NP	*In vitro*	Correlated findings with mRNA and protein levels.	Histone deacetylation may have a role in ECM production in human NP cultures and HDAC inhibitors may have a role as a therapeutic agent in NPs.	TGF beta-1 and ECM, alpha-SMA, fibronectin, collagen type I
3	Duan et al. (34)	eCRSwNP: 15; neCRSwNP: 14; Controls: 12	China	Nasal biopsy (unspecified) and ALI cultures from HNECs.	Both	HDAC inhibitor (JNJ-26481585) addition to ALI cultures increased transepithelial electrical resistance in CRS but not in controls.	Increased expression of HDAC1 and HDAC9 in eCRSwNP more than neCRSwNP. Treatment of eosinophilic CRSwNP ALI-HNECs with an HDAC inhibitor improved the tight junction expression and epithelial barrier integrity.	Role of HDAC inhibitors on epithelial integrity and tight junctions
4	Park et al. (35)	*In vitro* only	Korea	HNECs and PNECs cultured from ITM samples.	*In vitro*	Expression levels of HDAC2, HDAC4, and EMT markers E-cadherin, vimentin, fibronectin, SMA (alpha smooth muscle actin).	Trichostatin (an HDAC inhibitor) inhibits TGF beta-1-induced EMT.	Role of HDAC inhibitor on EMT and TGF beta-1

ALI, air–liquid interface; CRSwNP, chronic rhinosinusitis with nasal polyps; eCRSwNP, eosinophilic chronic rhinosinusitis with nasal polyps; ECM, extracellular matrix; EMT, epithelial–mesenchymal transition; HDAC, histone deacetylase; HNECs, human nasal epithelial cells; ITM, inferior turbinate mucosa; mRNA, messenger RNA; neCRSwNP, non-eosinophilic chronic rhinosinusitis with nasal polyps; NP, nasal polyps; NPDFs, nasal polyp-derived fibroblasts; PNECs, primary nasal epithelial cells; TSA, trichostatin A; CRS, chronic rhinosinusitis.

**Table 3:** Studies evaluating the role of alternative polyadenylation in chronic rhinosinusitis.

No.	Author/year	Subjects	Country of study	Tissue analyzed	*Ex vivo*/*in vitro*/both	Validation	Broad results	Biological pathway identified
1.	Tian et al. (36)	CRSwNP: 2	China	NP and matched UP mucosa	*Ex vivo*	Validation using RT-PCR	Switching of 3′ UTR lengths in NPs compared with UP. APA-mediated regulation of gene expression may play an important role in CRSwNP.	Wnt signaling (important pathway for immune cell maintenance and renewal) transcription, nucleolus, transcription regulation, mRNA splicing, apoptosis, protein modification, mitochondrion.
2	Tian et al. (37)	neCRSwNP: 13	China	NP and matched UP mucosa	*Ex vivo*	Validation using RT-PCR	Significant alteration in the tandem 3′ UTR length in 1,920 genes in NP samples. APA-mediated alternative 3′ UTR regulation plays a vital role in regulation of gene expression in these patients.	Transcription, protein targeting, transport localization, RNA splicing, mRNA metabolic process.

APA, alternative polyadenylation; CRSwNP, chronic rhinosinusitis with nasal polyps; neCRSwNP, non-eosinophilic chronic rhinosinusitis with nasal polyps; NP, nasal polyps; RT-PCR, reverse transcription polymerase chain reaction; UP, uncinate process; UTR, untranslated region.

**Table 4:** Study investigating genome-wide chromatin accessibility (ATAC-seq).

No.	Author/year	Subjects	Country of study	Tissue analyzed	*Ex vivo*/*in vitro*/both	Validation	Broad results	Biological pathway investigated
1.	Ordovas-Montanes et al. (38)	CRSwNP: 3; CRSsNP: 7	United States	NPs in CRSwNP and ethmoid sinus tissue in CRSsNP	*Ex vivo*	Confirmed by gene expression	Polyp basal cells were enriched in transcription factor motifs associated with undifferentiated state, chromatin opening, and oncogenesis.	Type 2 inflammation

ATAC-seq, assay for transposase-accessible chromatin using sequencing; CRSwNP, chronic rhinosinusitis with nasal polyps; CRSsNP, chronic rhinosinusitis without nasal polyps; neCRSwNP, non-eosinophilic chronic rhinosinusitis with nasal polyps; NPs, nasal polyps.

### DNA methylation studies

The number of CRS subjects in the studies ranged from 3 to 187. Eleven of 13 studies used a control arm with subject numbers ranging from 3 to 57. The majority (including the genome-wide studies) used inferior turbinate mucosa (ITM) as a control tissue; however, one study used posterior ethmoid (30), one used middle turbinate mucosa (20), one used uncinate process, and one used “normal nasal tissue” (19) as controls. Eight of the 13 studies looked at *in vivo* changes in nasal polyps (NPs), two looked at *in vitro* changes, and three studies investigated both *in vivo* and *in vitro*. The *in vitro* studies extracted human nasal epithelial cells (HNECs) from the NP tissue to prepare air–liquid interface cell cultures. Geographically, most of these studies were from the Asian populations (China, Korea, and Japan), one from the United States, and one from Europe (Belgium). For additional details, please refer to Table 1.

Six genome-wide DNA methylation studies found differences in global methylation between CRSwNP and controls (19–22, 26, 28, 31), while others that specifically investigated *TSLP* (23) (a cytokine involved in dendritic cell maturation and allergic inflammation), *IL-8* (24) (proinflammatory cytokine and a potent chemotactic factor, rapidly induced by bacterial and viral products), and *PLAT* (27) (encodes for a protein that converts the proenzyme plasminogen to plasmin, a fibrinolytic enzyme) found significant differences in methylation of CpG sites of these genes.

The first genome-wide DNA methylation study was published by Cheong et al. in a Korean population and compared CRSwNP with aspirin-intolerant asthma (AIA) and CRSwNP with aspirin-tolerant asthma (ATA) (31). Significant differences were found in methylation of interleukin (IL) genes, IL receptor genes, and genes involved in the arachidonic acid metabolism. *IL5RA, IL20RA, IL22RA*, and *PTGES* were hypermethylated in CRSwNP with AIA, and IL2RA, *IL10* and *CD28, PGDS, ALOX5AP,* and *LTB42* hypomethylated, as compared with those with CRSwNP with ATA (31). Zheng et al. identified that the *COLI8A1* gene was hypermethylated in CRSwNP vs. controls (28). However, this did not correlate with gene expression levels and immunohistochemistry. Kim et al. found hypermethylation in promoter regions of 10 genes and hypomethylation in 30 genes in CRSwNP compared with controls. They selected four genes for validation (*KRT19, NR2F2, ADAMTS1*, and *ZNF222*) and found that only some correlated with mRNA expression levels (26). Another genome-wide study investigated differential methylation in eosinophilic CRSwNP (eCRSwNP) vs. non-eosinophilic CRSwNP (neCRSwNP), and also compared them with controls. On comparing with controls, each group showed more than 500 differentially methylated genes, but on comparing with each other, only four were hypermethylated and 19 hypomethylated in eCRSwNP vs. neCRSwNP. FZD5 (a protein that is a receptor for the Wnt5A ligand) was significantly hypomethylated in the eCRSwNP, and the mRNA level of FZD5 was higher in the eCRSwNP than in the neCRSwNP group (22). Another group of investigators that studied eCRSwNP, neCRSwNP, and controls found differences in hydroxymethylated DNA levels. They found 26 differentially hydroxymethylated genes (DhMGs) between eCRSwNP and neCRSwNP, 169 DhMGs in eCRSwNP and controls, and only one DhMG between neCRSwNP and controls. They also found that the *GNAL* (encodes a stimulatory G protein alpha subunit that mediates odorant signaling in the olfactory epithelium), *INPP4A* (encodes an enzyme in the inositol metabolism pathway), and IRF4 [interferon (IFN) regulatory factor and regulation of interferon-inducible genes] expression levels were significantly different between eCRSwNP and the other two groups. The middle turbinate mucosa was the control tissue used, as opposed to ITM that was used by others (20). Kim et al. also found 44 differentially methylated probes (DMPs) in eCRSwNP including *IL13* (21). Some studies investigated the role of DNA methyltransferase (DNMT) and DNMT inhibitors. Global DNA methylation and DNMT activity were significantly increased in CRSwNP vs. controls and in CRSsNP vs. controls, along with an increased tissue expression level of DNMTs. In addition, nasal epithelial cells were cultured, and TGF beta-1 was shown to induce DNMT activity, global DNA methylation, and mRNA and protein levels of DNMT1, DNMT3A, and DNMT3B, after these had been knocked down using siRNA transfection. The authors also demonstrated that 5-azacytidine, a DNMT inhibitor, inhibited TGF beta-1- induced DNMT activity and epithelial–mesenchymal transition (EMT) in primary HNECs and air–liquid interface cultures (19). Shin et al. also demonstrated the use of DNMT inhibitors as potential therapeutic agents. They demonstrated that expression levels of *MeCP2*, a transcription regulator, were higher in NP vs. control ITM and was suppressed by 5-azacytidine, which had an inhibitory effect on TGF beta-1 nasal polyp-derived fibroblasts (NPDFs) and extracellular matrix (ECM) production (25).

*PLAT* was found to be hypermethylated in NP tissue of CRSwNP compared with control ITM from the same group of patients. Gene expression levels were also decreased in NP tissue. The authors suggested that this could possibly lead to excessive fibrin deposition and an aberrant coagulation cascade (27). Li et al. found that methylation levels at CpG sites in the proximal promoter region of *IL-8* were significantly decreased in NP of CRSwNP vs. ethmoid tissue of CRSsNP and ITM of control subjects, and this also correlated with IL-8 levels on ELISA, which were higher in CRSwNP vs. CRSsNP and controls (24). It has been found that despite the geographic diversity in the TH cytokine profiles across the globe, IL-8 levels were significantly upregulated in all population groups (39). *TSLP* showed hypermethylation in CRSwNP in two of 17 CpG units, as compared with CRSsNP and controls. However, gene expression or protein levels were not analyzed (23).

Only two DNA methylation studies originated outside of Asia (29, 30), of which one was an *in vitro* study (29). Seiberling et al. from the United States investigated and found elevated levels of modified forms of cytosine (5BrC and CIC), which are by-products of activated neutrophils and eosinophils and can result in an aberrant methylation of cytosine. The authors used posterior ethmoid tissue in controls and NP in CRSwNP (30). Pérez-Novo et al. from Belgium sought to investigate pathogen-induced methylation changes in NPs cultured with Staphylococcus enterotoxin B (SEB). They found that after 24 h of incubation, 43 genes exhibited changes in the methylation state, with 33 being hypermethylated and 10 hypomethylated. The two genes highlighted in their data analysis were *IKBKB* (activator of NF-kappa-B) and *STAT-5B* (member of the STAT family of transcription factors). Both play a crucial role in T-cell maturation/activation and immune response (29). Microbes and microbial dysbiosis have long been implicated in CRS etiopathogenesis, and further studies are needed to investigate pathogen-induced epigenetic changes. Similarly, smoking can lead to epigenetic changes as has been demonstrated in published literatures, but such studies are lacking in CRS.

To validate results, mRNA levels of some differentially methylated genes were investigated in 6 out of 13 studies and found to be generally consistent. A few studies also correlated protein expression using Western blotting or immunohistochemistry.

### Non-coding RNA studies

Out of the 44 studies investigating the role of non-coding RNAs, most studies investigated miRNAs, and only a few investigated other RNA types such as lncRNAs (40–44), siRNAs (45), and circRNAs (46, 47). Six studies used pre-existing GEO data sets to identify transcriptome-wide miRNA or lncRNA profiles of CRSwNP vs. controls, either exclusively or in conjunction with *in vivo* studies on CRS subjects (40–42, 44, 46, 48). Most studies had control subjects. The number of CRSwNP subjects in studies ranged from 4 to 164, and number of controls ranged from 3 to 50 subjects. A few studies investigated peripheral blood dendritic cells (DCs) (49–51), but most investigated sinonasal tissue (majority used NP in CRSwNP), and one study (the only pediatric epigenetic study) used NPs, serum, and eosinophils (52). For control sinonasal tissue, most used ITM. Only five studies originated outside of Asia. Refer to Supplementary Table S1 for additional details.

Studies either compared transcriptome-wide miRNA profiles (some of which additionally looked at specific miRNAs and their target genes), or only looked at specific miRNA levels [such as miR-19a (50), miR-150-5p (51), miR-124 (53), miR-663 (52), mir-142-3p (54), miR-21 (42), and miR-21-5p (55)] and their potential target genes. A total of 18 out of 44 studies looked at *in vivo* changes, 4 were only *in vitro*, 19 were both, and 3 exclusively performed a bioinformatic analysis on pre-existing GEO data sets. One study was exclusively done in a murine model of CRS (56), whereas five studies looked at murine models in addition to human subjects (42, 57–60). Overall, differences in miRNA and lncRNA profiles were found between CRSwNP and controls (40–44, 46, 47, 49, 61, 62, 63–66). The specific miRNAs that were investigated found some of the target genes and pathways as follows: IL-10 (67, 68), IL-5 (64), IL-6 (69), aryl hydrocarbon receptor (AHR) (transcription factor that also modulates immune response) (53), *EGR2* (51), TGF beta-1 (52, 62, 70–73) and tumor necrosis factor (TNF) alpha (54, 60), glucagon-like peptide-1 and IL-33 (55), EGF (74), IRF4 (42), PI3K/AKT pathway (40, 56, 58, 65, 75), mucin secretion (76), MMP (77), EMT, VE-cadherin (78), and ROR alpha (61).

Zhang et al. compared global miRNA expression levels between eCRSwNP, neCRSwNP, CRSsNP, and controls. Distinct miRNA profiles were seen in eCRSwNP and CRSsNP. MiR-125b was upregulated in CRSwNP, and the authors showed that miR-125b inhibitor decreased interferon-alpha *in vitro* and IFN-beta *in vivo*. Further, IFN-β expression was increased in eosinophilic CRSwNP, and IFN-β mRNA levels positively correlated with IL-5 mRNA levels and eosinophil infiltration in sinonasal mucosa. They concluded that upregulated miR-125b can enhance type I interferon expression in airway epithelial cells (63). Though in another study that compared eCRSwNP with neCRSwNP, CRSsNP, and controls, no differences in miRNA expression were found (79). Luo et al. studied dendritic cells from peripheral blood in CRSwNP vs. controls and found lower IL-10 expression and higher miR-19a expression in subjects and concluded that miR-19a plays a role in the suppression of IL-10 in peripheral dendritic cells. They also found that knocking down the *miR-19a* gene could abolish the effect of recombinant IL-4-induced suppression of IL-10 expression (50). Ma et al. also studied dendritic cells from peripheral blood and found upregulated expression of miR-150-5p in CRS. They also found that its target gene was *EGR2* (early growth response 2) in DCs (80). These authors had previously compared and found distinct miRNA expression patterns in dendritic cells from peripheral blood in atopic CRSwNP, non-atopic CRSwNP, and CRSsNP as compared with controls, with 31 commonly changed miRNAs in all CRS subjects (49).

Liu et al. found that AHR, which is essential for modulating the immune response, was significantly higher in NPs and had an inverse correlation with the expression changes of miR124 (53). Qing et al. in 2021 suggested the role of miR-142-3p in the body's inflammatory response through the TNF alpha signaling pathway in CRSwNP. They studied ITM in subjects as well as controls (54). Liu et al. found miR-21 higher in NPs of CRSwNP vs. controls but no difference between CRSsNP and controls. MiR-21 also positively correlated with IL-10 and negatively with IL-1 beta, IL-6, IL-8, IL-33, TSLP, and Lund Mackay and Lund Kennedy scores. They concluded that miR-21 could be a prominent negative feedback regulator in the inflammatory process (67). Li et al. identified six differentially expressed miRNAs between CRSwNP and CRSsNP, one (miR-4492) between CRSwNP and controls, and none between CRSsNP and controls. They demonstrated that miR-4492 is downregulated, and IL-10 is upregulated in NPs. Using a bioinformatic analysis, they predicted its targets genes as IL-10 and TNF alpha. They found elevated levels of IL-10 in CRSwNP and a negative correlation of miR-4492 and IL-10 but no correlation with TNF alpha (68). Luo et al. had found lower IL-10 expression in peripheral blood dendritic cells of CRSwNP (50).

Xuan et al. found 25 differentially expressed miRNAs between CRSwNP and controls. On pathway analysis, they found that upregulated miRNAs were involved in mucin type O-glycan biosynthesis, and downregulated miRNAs were involved in the TGF beta-1 signaling pathway (62). Another study found the miRNA transcriptome related to ciliogenesis and ciliary function impaired in during differentiation of CRSwNP nasal epithelium as compared with controls (81). Other non-coding RNA studies have also implicated dysregulation in ciliogenesis (81), mucin secretion (76), or mucin type-O-glycan biosynthesis (44, 65, 66).

Yu et al. performed the only pediatric epigenetic study in CRSwNP and compared 35 CRSwNP with 46 controls. In addition to NP (in CRSwNP) and ITM (in controls), they also studied serum and peripheral eosinophils in both groups. They found TGF beta-1 mRNA and protein to be higher in all types of samples from subjects and decreased miR-663 expression in NPs and eosinophils. They concluded that miR-663 may be a negative regulator of TGF beta-1 in children with NPs (52). Several other miRNA studies have implicated or investigated the role of EMT and/or TGF beta-1 (48, 65, 70–73, 75, 82).

Other pathways investigated or identified in these studies include those related to T cells and cytokines (40, 43, 48, 55, 60, 66), eosinophil trafficking (59), PI3K/AKT signaling (40, 56, 58, 65, 75), Ras–MAPK signaling (47, 66, 69, 75), Wnt signaling (47, 57), ECM (83, 84), adherens junctions (65, 83, 84), focal adhesions (47, 65, 84), matrix metalloproteinases (77), vascular permeability (45, 78), cell cycle and apoptosis (64, 85), EGF (74), ROR alpha (on exposure to pollution) (61), and IRF4 (42).

### Histone modification studies

There are only four studies on the role of histone modifications, and all have looked at histone deacetylation. All performed *in vitro* experiments using NPDFs or HNEC cultures. Kindly refer to Table 2 for additional details.

Both studies by Cho et al. on NPDFs illuminate the potential role of trichostatin A (TSA), a histone deacetylase (HDAC) inhibitor, for reversing TGF beta-1-induced ECM accumulation. In their first study, they demonstrated that TSA treatment reversed the morphological changes, ECM accumulation, and myofibroblast differentiation in NPDFs that were stimulated with TGF beta-1. They correlated their findings with mRNA expression and found that TSA reduced alpha-SMA and collagen at mRNA and protein level. They also demonstrated elevated HDAC2 levels in NP vs. ITM tissue and that TSA inhibited HDAC levels. Using histone levels, they also demonstrated that TSA leads to hyperacetylation (32). In their subsequent study, the authors also demonstrated that TSA inhibited TGF beta-1-induced changes in NPDFs, and this was done through the suppression of protein expression of HDAC2 and HDAC4 (33). The other studies by Duan et al. (34) and Park et al. (35) also investigated the use of HDAC inhibitors. Duan et al. similarly found increased expression of HDAC1 and HDAC9 in nasal biopsy specimens of eCRSwNP as compared with neCRSwNP (34). All these studies have found a potential role of these drugs in treating CRSwNP. With the availability of HDAC inhibitors as therapeutic agents in other diseases, new possibilities for treatment of CRS using HDAC inhibitors may be tapped in the future.

### APA studies

Only two studies on the role of APA in CRS were found, both by the same authors from China. Both looked at *in vivo* changes and used matched control tissue from the uncinate process mucosa (UPM). Sample sizes were small, with two CRSwNP subjects in the first study and 13 non-eosinophilic CRSwNP patients in their second study. They have shown significant differences in 3′ UTR lengths in NPs compared with control tissue. The pathways that were enriched in these genes were those related to regulation of transcription, macromolecule catabolic localization, and mRNA processing (36, 37). Refer to Table 3 for additional details.

### Chromatin accessibility

Ordovas-Montanes studied chromatin accessibility in their transcriptomic study in CRS. They found that polyp basal cells were enriched in transcription factor motifs associated with undifferentiated state, chromatin opening, and oncogenesis. This study was primarily an RNA-sequencing study but additionally performed an epigenetic profiling using the assay for transposase-accessible chromatin using sequencing (ATAC-seq) profiling (38). Refer to Table 4 for details.

## Discussion

The studies identified in the systematic review were heterogeneous in sample sizes, type of tissue analyzed, control tissue used, and epigenetic mechanism studied. Interestingly, a common theme of principal biological pathways involved in inflammation, cytokine signaling, type 2 response, tissue remodeling, structural integrity, arachidonic acid metabolism, ciliary function, coagulation, and transcription were noted on these studies. In a recent comprehensive review on the role of genetics and epigenetics in CRS, Lal et al. also identified several genes and pathways in CRS that were common in both genetic and epigenetic studies (17). Therefore, the familial predisposition to CRS that has been noted (14, 86) may be a result of genetic factors, epigenetic factors, or both. Epigenetics in CRS has been investigated through different epigenetic mechanisms, but the most commonly investigated is the role of miRNAs followed by DNA methylation, as was also noted by Li et al. in a recent review (18).

The role of TGF beta-1 was identified or investigated through almost all epigenetic mechanisms. It plays an important role in tissue remodeling (potent stimulator of osteoblastic bone formation) and can also induce EMT. In addition, it can regulate cell proliferation, differentiation, and growth; immune function; and expression and activation of other growth factors including interferon gamma and tumor necrosis factor alpha. It promotes either T-helper 17 cells (Th17) or regulatory T cells (Treg) lineage differentiation in a concentration-dependent manner (87). The role of TGF beta-1 has been extensively investigated in asthma (87–90). In addition, its role as a biomarker in diagnosing severe asthma has also been investigated (91). TGF beta ligands have found to be upregulated in several lung conditions including asthma, pulmonary fibrosis, emphysema, and lung cancer (92). Park et al. demonstrated that TGF beta-1 induced DNMT expression and DNA methylation. However, 5-azacytidine (a DNMT inhibitor) was able to inhibit TGF beta-1-induced DNMT and EMT, thus having a potential therapeutic role in CRS (19, 25). In addition, miRNA studies were able to identify TGF beta-1 as a target of miR-663 in pediatric NPs (52). Several other adult miRNA studies have implicated or investigated the role of EMT and/or TGF beta-1 (48, 65, 70–73, 75, 82). Histone deacetylation studies revealed that HDAC inhibitors like trichostatin could block proliferation in TGF beta-1-induced NPDFs (32, 33, 35). In addition to epigenetic studies, genetic studies that have been replicated have also found polymorphisms in the *TGF beta-1* gene to be associated with CRS (93, 94) even after excluding asthma (94). Its role in pathophysiology of NPs has been studied in animal models as well (95). Both DNMT inhibitors and HDAC inhibitors have a promising role in targeting TGF beta-1-induced EMT in NPs.

Several interleukins and members of the cytokine family have also been investigated and identified in epigenetic studies. These include IL-10, IL-8, IL-1 beta, IL-5, IL-6, IL-33, IL2RA, IL5RA, IL20RA, IL22RA1, CD28, PIK3CG, TSLP, CCL18, IRF4, and type I interferons (23, 24, 31, 50). *IL-10* (a cytokine with pleiotropic effects in immunoregulation and inflammation that downregulates the expression of Th1 cytokines, enhances B-cell survival and proliferation, and antibody production) has been shown to be epigenetically regulated through DNA methylation (31), as well as micro-RNAs (50). It was shown to be hypomethylated in AIA, implying higher levels of IL-10 (31). In other studies related to miRNAs, its expression was reported as higher in NPs in two studies, and it was regulated bymiR-4492 (68) and miR-21 (67) and lower in one study and regulated by miR-19a (50). The promoter region of *IL-8* gene, a proinflammatory cytokine, chemotactic factor, and potent angiogenic factor, was significantly hypomethylated and was associated with significantly higher IL-8 expression levels (24). Other proinflammatory cytokines mentioned above include those with a predominant role against microbes (type I IFN, IL-6) and Th2 response (IL5RA, CCL18, TSLP, CD28, and IL22RA). TNF alpha, a potent proinflammatory cytokine, was also found to be higher in CRSwNP and also associated with higher miR-142-3p levels (54). Polymorphisms in *TNF alpha* gene have also previously been identified in geographically diverse populations in CRS (96–98). TNF alpha inhibitors are currently used to treat multiple inflammatory conditions such as rheumatoid arthritis, psoriasis, inflammatory bowel disease, and ankylosing spondylitis. They have been investigated *in vivo* for the treatment of asthma and chronic obstructive pulmonary disease (COPD) (99). Given the availability of drugs that target some of the key molecules implied in CRS pathophysiology, there is a great potential to develop novel therapeutics in CRS targeting these proteins. The pathways identified in non-coding RNA studies also relate to immune function, T cells and cytokines (40, 43, 48, 55, 60, 66), and eosinophil trafficking (59).

Dysregulations in ciliogenesis and ciliary function have also been identified. These include involvement of the mucin type-O-glycan biosynthesis pathway (44, 62, 65, 66, 81), mucin secretion (76), and identification of GALNT7 through a lncRNA study (44). Impairment of pathways and implication of genes involved in structural integrity include *COL18A1* (28), integrin genes (*ITGB2*, *ITGAL*, and *ITGAM*) (31), *KRT19* (26), alpha-SMA*,* fibronectin*,* and collagen type 1 (32, 33), and miRNA studies have found pathways involving adherens junctions (65, 83, 84) and focal adhesions (47, 65, 84). An impaired epithelial barrier is a commonly accepted pathophysiological mechanism in CRS, and whether the epigenetic modifications in genes involved in the nasal epithelial structure is a cause or consequence of the inflammatory process is yet to be discerned. Other pathways identified include those involved in coagulation. *PLAT* gene encodes for a serine protease that converts the proenzyme plasminogen to plasmin, a fibrinolytic enzyme. It participates in coagulation, cell migration, and tissue remodeling. Methylation levels in its promoter were significantly higher and its expression levels lower, possibly leading to the deposition of fibrin (27). Alterations in vascular permeability (45, 78) have also been associated with CRS through non-coding RNA studies. In addition, *ADAMST1* (anti-angiogenic activity and active metalloprotease) was hypomethylated, implying increase in angiogenic activity (26). An miRNA study also found that miR-29b-3p expression was positively correlated with the expression of MMP-2 and MMP-9 in CRSwNP (77).

One of the first epigenetic studies in CRS that compared AIA with ATA found aberrations in the arachidonic acid metabolism pathway. Hypomethylation in *PGDS* (a potent inhibitor of platelet aggregation), *ALOX5AP* (a protein that converts arachidonic acid to ALOX5), *LTB4R* (leukotriene B4 receptor involved in inflammatory response and the neuropeptide signaling pathway), and hypermethylation in *PTGES* [terminal enzyme of the cyclooxygenase (COX)-2-mediated prostaglandin E2 biosynthetic pathway] were identified (31).

Genes encoding for transcription factors, which can in turn regulate expression levels of other proteins, have also been identified. These include *MeCP2* (can bind to methylated DNA, transcription regulation, and gene expression) (25), *NR2F2* (ligand-inducible transcription factor that is involved in the regulation of many different genes) (26), *AHR* (transcription factor; among its related pathways are beta-2 adrenergic-dependent CFTR expression and adipogenesis) (53), *EGR2* (transcription factor, plays a role in peripheral nerve myelination, may play a role in adipogenesis) (51), and *FZD5* (receptor for Wnt5A, and its complex with Wnt5A contributes to activating inflammation and tissue modification) (22). Both APA studies found differences in pathways related to Wnt signaling (important pathway for immune cell maintenance and renewal) transcription, nucleolus, transcription regulation, mRNA splicing, apoptosis, and protein modification (36, 37). Non-coding RNA studies have also found differences in Wnt signaling (47, 57) and cell cycle and apoptosis (64, 85). Other pathways like PI3K/AKT signaling (40, 56, 58, 65, 75) and Ras–MAPK signaling (47, 66, 69, 75), which are important in cell cycle regulation, have also been identified in non-coding RNA studies.

### Limitations

Our study has limitations. Due to the heterogeneity of sample sizes, methodology, and control tissue utilized, comparing studies with each other or drawing common conclusions is challenging. In addition, epigenetics may vary based on race, ethnicity, and geographical location, and since most of these studies have been performed in Asia, it may be difficult to generalize the findings across other populations. Just as genetic differences between races can affect the interpretation of genetic studies in CRS from different parts of the world, similarly geographical and environmental differences can lead to different epigenetic changes. Type 2 endotype is more common in patients with NPs in the western world vs. Asians, where mixed endotype remains dominant (39). In addition, most studies classify CRS based on phenotype (CRSwNP and CRSsNP); however, there is emerging evidence, including transcriptomic data, to show that polyp status may not reveal true pathogenetic mechanisms at play (100). This makes it imperative to study genetic and epigenetic mechanisms across racially and geographically diverse populations. The presence of co-morbid conditions like asthma and allergic rhinitis may influence results of studies, and this may limit our understanding of CRSwNP-specific epigenetic changes. Bioinformatic analyses on pre-existing GEO data sets need to be consistent in including studies that have used the same disease and control tissue, and this has not been done uniformly across these studies (40–42). In a recent review on epigenetics in CRS, authors have elaborated that several miRNA studies have shown controversial results in whether a specific miRNA was upregulated or downregulated in CRS (18). Because of the conflicting data, the interpretation of epigenetic studies in CRS remains a challenge.

### Areas of further research

Epigenetic studies on racially and geographically distinct populations are the need of the hour. In addition, studying the epigenetic effects of different environmental stimuli, including different microbes, smoking, and the effects of air pollution (performed *in vitro* by one study) (61) may lead to further insights on disease etiopathogenesis, as may longitudinal studies. Further research also needs to account for confounding factors such as asthma and allergic rhinitis, the presence of which may make interpretation of results more complicated. Studies on pediatric age groups have also not been undertaken, barring one miRNA study from China (52).

Since epigenetic modifications are reversible, and greater research on developing targeted therapeutics using HDAC inhibitors and DNMT inhibitors may lead to a paradigm shift in the treatment of the subsets of CRS.

In addition, existing data sets of transcriptomic studies in CRS from different regions of the world can be used to identify and compare non-coding RNA profiles across diverse populations; however, caution in matching disease and control tissue should be exerted.

## Conclusions

Differences in methylation patterns, non-coding RNAs, histone deacetylation, APA, and chromatin accessibility studies suggest that in CRS subjects, there is likely a major impact of the environment. Overall, the main genes and biological pathways implicated were those involved in inflammation, cytokine signaling, type 2 response, tissue remodeling, arachidonic acid metabolism, ciliogenesis and mucin secretion, coagulation, and regulation of transcription. Since epigenetic changes are pharmaceutically reversible, it is possible that future studies can be leveraged to develop targeted therapeutics in CRS.

## Data Availability

The original contributions presented in the study are included in the article/Supplementary Material, further inquiries can be directed to the corresponding author.
