# Supplementary material for: Insights into the epigenetics of chronic rhinosinusitis with and without nasal polyps: a systematic review
Source: Front Allergy. 2023 May 22;4:1165271. doi: 10.3389/falgy.2023.1165271 (PMC10240395; doi:10.3389/falgy.2023.1165271)
Supplement: Supplementary file 1 [file Table1.docx]

**Supplementary Table 1**: **Studies evaluating the role of Non-coding RNAs in Chronic rhinosinusitis**

| **No.** | **Author/ Year** | **Subjects or GEO datasets used** | **Country of study** | **Tissue analyzed** | **In vivo/ in vitro/Both** | **Validation** | **Broad results** | **Specific Genes/ Biological pathway identified or investigated** |
| --- | --- | --- | --- | --- | --- | --- | --- | --- |
| 1 | Mimmi et al/ 2022 (77) | CRSwNP pre and post Dupilumab- 4 | Italy | Serum | In vivo | - | Significant downregulation post dupilumab treatment only for hsa-miR-25-3p and hsa-miR-185-5p. 34 KEGG pathways were significantly enriched with genes representing targets of these miRNAs | Adherens junction and ECM-receptor interaction in the top 10 pathways |
| 2 | Chen et al/ 2022 (65) | CRSwNP-17; CRSsNP- 15; Controls- 19 | China | ‘Sinus mucosa’ in CRS;  Controls- ‘oral mucosa samples of maxillary sinus’ | In vivo and murine model | In mouse model with CRS, established the EGF HIF-1α–miR-21–AQP4 axis. | Compared with normal sinus mucosa, EGF, HIF-1α, and miR-21 were upregulated and AQP4 was downregulated in sinus mucosa from patients with CRS and in a CRS mouse model. | EGF regulates HIF-1α–miR-21–AQP4 axis |
| 3 | Luan et al/ 2022 (45) | eCRSwNP-20; neCRSwNP- 12; Controls- 16 | China | ‘nasal tissue samples’ | Both | Genes associated with type 2 inflammation were extensively downregulated at the transcriptome level in miR-21 knockout mice | miR-21-5p aggravates type 2 inflammation in the nasal mucosa of patients with CRSwNP via targeting glucagon-like peptide-1 receptor/IL-33 signaling, which may be a potential therapeutic target for CRSwNP. | Glucagon-like peptide-1 receptor/IL-33 signaling |
| 4 | Sun et al/ 2022 (36) | CRSwNP- 14; Controls- 14  Gene Expression Omnibus database  [GSE169375](https://www.ncbi.nlm.nih.gov/geo/query/acc.cgi?acc=GSE169375) (circRNA): China [GSE169376](https://www.ncbi.nlm.nih.gov/geo/query/acc.cgi?acc=GSE169376) (miRNA): China [GSE36830](https://www.ncbi.nlm.nih.gov/geo/query/acc.cgi?acc=GSE36830) (mRNA): USA | China | [GSE169375](https://www.ncbi.nlm.nih.gov/geo/query/acc.cgi?acc=GSE169375): Nasal mucosa;  [GSE169376](https://www.ncbi.nlm.nih.gov/geo/query/acc.cgi?acc=GSE169376): Nasal mucosa;  [GSE36830](https://www.ncbi.nlm.nih.gov/geo/query/acc.cgi?acc=GSE36830): NP for CRSwNP, UP for CRSsNP and controls; | Both | Verified by in-vitro cell experiments | Differential expression of 5423 circRNAs, 415 miRNAs, and 3673 mRNAs identified in CRSwNP compared to controls. hsa_circ_0031594, hsa-miR-1260b, and NCAPG2, and hsa_circ_0031594, hsa-miR-6507-5p, and PRC1 may be key regulators for CRSwNP occurrence. | - |
| 5 | Wang et al/ 2022 (30) | CRSwNP- 57, Controls-37  [GSE136825](https://www.ncbi.nlm.nih.gov/geo/query/acc.cgi?acc=GSE136825): Singapore  [GSE36830](https://www.ncbi.nlm.nih.gov/geo/query/acc.cgi?acc=GSE36830): USA  [GSE169376](https://www.ncbi.nlm.nih.gov/geo/query/acc.cgi?acc=GSE169376): China | China | [GSE136825](https://www.ncbi.nlm.nih.gov/geo/query/acc.cgi?acc=GSE136825): NP for cases, IT for controls; [GSE36830](https://www.ncbi.nlm.nih.gov/geo/query/acc.cgi?acc=GSE36830) NP for CRSwNP, UP for CRSsNP and controls;  [GSE169376](https://www.ncbi.nlm.nih.gov/geo/query/acc.cgi?acc=GSE169376): ‘Nasal mucosa’ for CRSwNP and Controls | In vivo | - | 48 miRNAs, 304 lncRNAs identified as CRSwNP specific. 5 biomarkers in CRSwNP, namely, LINC01198, LINC01094, LINC01798, LINC01829, and LINC01320 | Chemokine signaling pathway, PI3K-AKT signaling pathway |
| 6 | Song et al/ 2022 (47) | CRSwNP-37, CRSsNP-29 Murine model | China | NPs from CRSwNP and sinus tissue from CRSsNP | In vivo and murine model | Dual luciferase assay and mouse model of CRS | miR-125b is upregulated by Sp1 and induces inflammation and fibrin deposition in NPs by activating the Wnt/β-catenin signaling pathway. | Wnt/β-catenin signaling pathway |
| 7 | Morawska-Kochman et al/ 2022 (79) | CRSwNP- 10; Controls-10 | Poland | CRS: Maxillary sinus inflamed mucosa;  Controls: maxillary sinus mucosa | In vivo | - | CSRwNP showed increased levels of miR-203a-3p and decreased expression of miR-17-5p and miR-145-5p. Pro-apoptotic transcripts detected at mRNA and miRNA levels in CRSwNP. | Detection of markers associated with cell survival and apoptosis |
| 8 | Li et al/ 2022 (31) | eCRSwNP-12; Controls-12  GSE36830: USA GSE32300: China; GSE72713: China | China | GSE36830: NP for CRSwNP, UP for CRSsNP and controls; GSE32300: ‘Sinonasal mucosa’ from controls and CRS; GSE72713: NP from CRSwNP and sphenoid sinus mucosa from controls | - | - | Overall, a total of 964 DE-mRNAs, 358 DE-miRNAs, and 56 DE-lncRNAs were obtained.  There were six hub lncRNAs in the ceRNA network but only two lncRNAs (MSC-AS1 and CARD8-AS1) were mainly located in the cytoplasm. Among the two, MSC-AS1 displayed the largest network, suggesting its broad regulation effect of gene expression in eosinophilic CRSwNP. | - |
| 9 | Jian et al/ 2022 (48) | CRSwNP-36; CRSsNP- 25; murine model | China | NPs from CRSwNP and nasal mucosa from CRSsNP, Also murine model | In vivo and murine model | Confirmed in murine model | STAT1 acts as a transcription factor to promote transcription of miR-18a in CRSwNP. | The PI3K/AKT pathway was activated in the nasal polyp and regulated by the STAT1/miR-18a/PTEN axis. |
| 10 | He et al/ 2022 (78) | CRSwNP-5; Controls-5 | China | Plasma exosomes | In vivo | - | 159 exosomal miRNAs were differentially expressed by miRNA sequencing in CRSwNP. | Pathways: axon guidance, extracellular matrix (ECM)-receptor interaction, protein digestion and absorption, the calcium, the Hippo, the Notch, the ErbB, the cAMP signaling pathway, and focal adhesion |
| 11 | Chen et al/ 2022 (32) | CRSwNP-71; Controls-44  GSE136825: Singapore GSE36830: USA GSE179265: USA and GSE169376: China | China | GSE13682535: NP in CRSwNP; IT in controls; GSE36830: NP for CRSwNP, UP for CRSsNP and controls; GSE 179265: NP for CRSwNP, UP for CRSsNP and controls; GSE169376: ‘Nasal mucosa’ for CRSwNP and Controls | In vivo | - | 565 DE-lncRNAs, 23 DE-miRNAs, and 1799 DE-mRNAs. MIAT/miR-125a/IRF4 axis may play a critical role in the development and progression of CRSwNP. | MIAT/miR-125a/IRF4 axis |
| 12 | Qing et al/ 2021(44) | CRSwNP-20; Controls-20 | China | ITM in all patients | Both | Dual luciferase assay and mRNA levels | miR-142-3p and TNF-alpha were significantly higher in nasal mucosa of CRSwNP. miR-142-3p may play a role in the body’s inflammatory response through TNF-alpha signaling pathway in CRSwNP. | miR-142-3p and TNF-alpha (multifunctional proinflammatory cytokine, regulation of a wide spectrum of biological processes including cell proliferation, differentiation, apoptosis, lipid metabolism, and coagulation) |
| 13 | Liu et al/ 2021(58) | CRSwNP- 45; CRSsNP-10; Controls-15  Nasal epithelial cells by brushings of 10 controls and 17 NP | China | NP from CRSwNP, UP from CRSsNP, Turbinates from controls and nasal epithelial cells brushings from nasal mucosa were collected. | Both | Confirmed by in-vitro experiment on HNEC cultures | MiR-21 higher in CRSwNP versus controls but no difference between CRSsNP and controls.  MiR-21 positively correlated with IL-10 and negatively with IL-1 beta, IL-6,IL-8, IL-33, TSLP and Lund Mackay and Lund Kennedy scores.  MiR-21 could be a prominent negative feedback factor in the inflammation process and may have anti-inflammation role in CRSwNP. | MiR-21higher and positively correlated with IL-10 and negatively with IL-1 beta, IL-6,IL-8, IL-33, TSLP |
| 14 | Zhang et al/ 2021 (75) | CRSwNP-30; Controls- 10 | China | NP from CRSwNP and IT from controls | Both | Validated in in vitro model using HNECs | miR-30a-5p was down-regulated, with alteration of EMT markers expression in NPs.   CDK6 was validated as a direct target of miR-30a-5p. miR-30a-5p could attenuate EMT via repressing the expression of the CDK6 | EMT, TGF beta-1 |
| 15 | Zhang et al/ 2021 (60) | CRSwNP- 20 | China | NP from CRSwNP and IT of subjects used as controls | Both | Validated in NPDFs | Let-7a-5p was significantly downregulated in CRSwNP tissues and cells. IL-6 was found to be a target gene of let-7a-5p. | Ras-MAPK pathway |
| 16 | Yu et al/ 2021 (37) | eCRSwNP-3; neCRSwNP-3; Controls-3 | China | NP from CRSwNP and MT from controls | In vivo | Some were validated using qRT- PCR | In the CRSwNP group, 1794 circRNAs were downregulated and 1,081 circRNAs were upregulated, the expression of 192 miRNAs was significantly downregulated, and none of the miRNAs were significantly upregulated. | Differential circRNAs were enriched in amoebiasis, salivary secretion, cell adhesion molecules actin cytoskeleton etc and and miRNAs in pathways in cancer, endocytosis, thyroid hormone signaling, focal adhesion, MAPK signaling, wnt signaling etc. |
| 17 | Jiang et al/ 2021 (64) | eCRSwNP- 26; neCRSwNP- 23; Controls- 15 | China | NP in CRSwNP;  IT in controls | Both | TGF-β1 upregulated miR-182 expression and promoted EMT in hNEPCs. these upregulations were reversed by miR-182 inhibitor. | miR-182-induced EMT in response to TGF-β1 might promote nasal polyp genesis in both Eos CRSwNP and non-Eos CRSwN. TGF-β1 upregulated miR-182 expression and promoted EMT in hNEPCs. these upregulations were reversed by miR-182 inhibitor. | EMT, TGF beta 1 |
| 18 | Silveira et al/ 2021 (55) | CRSwNP- 36; Controls- 41 | Brazil | NP from CRSwNP and MT from controls | In vivo | Validated by qPCR | six microRNAs were up-regulated in CRSwNP samples when compared with controls: miR-205-5p, miR-221-3p, miR-222-3p, miR-378a-3p, miR-449a and miR-449b-5p. miR-205-5p showed a significantly positive correlation with IL-5 concentration and eosinophil count | Cell cycle regulation and apoptosis |
| 19 | Shin et al/ 2021 (63) | In vitro only | Korea | HNECs | In vitro | Verified using protein levels by immunofluorescence staining | miR-29b down-regulates TGF-β1-induced EMT and cell migration via HSP47 in airway epithelial cells. | EMT and TGF beta 1 |
| 20 | Liu et al/ 2021 (68) | CRSwNP-100 | China | NPs, HNECs | Both | Dual‑luciferase assay revealed that miR‑29b‑3p targeted the 3' untranslated region of MMP‑2/MMP‑9 | miR-29b-3p expression was positively correlated with the expression of MMP-2 and MMP-9 in CRSwNPs, and TIMP-1 expression was negatively correlated with the expression of MMP-2 and MMP-9. miR-29b-3p affects the acetyl-α-tubulin levels by increasing MMP-9 and integrin β1 interaction. | MMP (Matrix metalloproteinases) |
| 21 | Hao et al/ 2021 (38) | CRS-6; Controls-3  [GSE169376](http://www.ncbi.nlm.nih.gov/geo/query/acc.cgi?acc=GSE169376): China for screening miRNAs | China | GSE169376: ‘Nasal mucosa’ for CRSwNP and Controls. HNECs for in vitro experiment | In vitro | - | miR-1287-5p was downregulated in CRS patients and LPS-induced HNECs. upregulation of miR-1287-5p could inhibit HMGB1 and SNAI1, thus curbing the pro-inflammatory cytokines, IL-6, IL-8 and TNF-α and EMT process. | EMT and pro-inflammatory cytokines |
| 22 | Cha et al/ 2021 (56) | CRSwNP-7; CRSsNP- 8; Controls- 7 | Korea | Extracellular vesicles in Nasal lavage fluid | In vivo | - | Five upregulated and seven downregulated miRNAs were differentially expressed in the NLF-EVs of CRS patients compared to controls, while eight upregulated miRNAs were differentially expressed in the NLF-EVs of CRSwNP versus CRSsNP. | Mucin-type O-glycan biosynthesis, Hippo signaling pathway, FoxO signaling pathway, PI3K-Akt signaling pathway, focal adhesion, adherens junction, Rap1 signaling pathway, and transforming growth factor-beta (TGF-β) signaling pathway were the most prominent pathways |
| 23 | Bu et al/ 2021 (57) | eCRSwNP-10; neCRSwNP-5; Controls-9 | China | NP from CRSwNP and IT from controls | In vivo | miRNAs of interest and their target genes were validated using quantitative real-time polymerase chain reaction (PCR | 71 DE-miRNAs (38 upregulated and 33 downregulated microRNAs) in eCRSwNP patients vs control subjects, 188 DE-miRs (106 upregulated and 82 downregulated mRNAs) in neCRSwNP patients vs control subjects, and 119 DE-miRNAs (54 upregulated and 65 downregulated mRNAs) in neCRSwNP vs ECRSwNP patients. Overall, 31 common DE-miRNAs were shared by eCRSwNP patients vs control subjects and non-eCRSwNP patients vs control subjects | Mainly associated with mucin-type O-glycan biosynthesis, MAPK signaling pathway, cytokine-cytokine receptor interaction, and Rap1-signaling pathway |
| 24 | Zhang et al/ 2020 (69) | CRSwNP-26; Controls-10 | China | Exosomes from Nasal lavage fluid (NLF) and Human umbilical vein endothelial cells (HUVECs) | Both | miR-22-3p regulated the vascular permeability by targeting VE-cadherin in HUVECs. | miR-22-3p was upregulated in NLF-derived exosomes from CRSwNP. Vascular endothelial- (VE-) cadherin (CDH5) was identified as a direct target of miR-22-3p. | Vascular permeability |
| 25 | Yang et al/ 2020 (62) | CRSwNP-11; CRSsNP-14; Control-10 | China | NPs from CRSwNP, MT from CRSsNP and IT from controls and HNECs | Both | TGF-β1 upregulates miR-155-5p expression and induces EMT in HNECs, which is reversed by miR-155-5p inhibitors | miR-155-5p was significantly increased in the CRSwNP and CRSsNP groups compared with the control. miR-155 inhibitors may be a novel anti-polyp drug | EMT, TGF beta 1 |
| 26 | Yan et al/ 2020 (67) | CRSwNP-15; CRSsNP-15; Controls-15 | China | NPs from CRSwNP; UP from CRSsNP and controls and HNECs | Both | On dual luciferase reporter assay, it was confirmed that EGFR is a target gene of miR-146a. | miR-146a acted on EGFR gene and inhibited its activation, thereby downregulating MUC5AC expression | Mucin secretion |
| 27 | Wang et al/ 2020 (33) | CRSwNP + asthma- 65; CRSwNP- 99; Controls- 31 | China | NPs from CRSwNP and IT from controls | In vivo | - | 176 common DE-lncRNAs shared by CRSwNP + AS versus control and CRSwNP-alone versus control. LINC01146 as hub lncRNA dysregulated in both subtypes of CRSwNP | LINC01146 was mostly associated with T cell receptor signalling pathway, natural killer cell mediated cytotoxicity, Fc gamma R-mediated phagocytosis, and Th1 and Th2 cell differentiation |
| 28 | Shin et al/ 2020 (51) | CRS-9; Controls-9 | Korea | NP for CRS and IT for controls and primary HNECs | In vitro, on exposure to PM (pollution) | Similar findings in patients of CRS, as obtained in HNECs on exposure to PM | 58 significant differentially regulated miRNAs in PHNECs upon PM exposure. MiR-19a and miR-614 were highest and predicted RORα as a target of these. Enhanced expression of miRNA-19a and miRNA-614 but reduced RORα expression in a CRS patient tissue compared with the normal | Inflammatory, RORα expression |
| 29 | Callejas-Dfaz et al/ 2020 (73) | CRSwNP-7; Controls-7 | Spain | NP from CRSwNP, ITM from controls | In-vitro | Confirmed by mRNA on RT-PCR | Transcriptome-wide analysis of mRNA and mi-RNA done during in vitro mucociliary differentiation of human adult basal stem cells from NP and ITM found that the transcriptome related to ciliogenesis, and ciliary function is significantly impaired during differentiation of CRSwNP epithelium, due to an altered expression of miRNAs. | Ciliogenesis and ciliary function is significantly impaired |
| 30 | Korde et al/ 2020 (49) | CRS-40 and an Asthma cohort and mouse model of CRS | USA | Inflamed tissue from any of the sinuses in CRS and sputum and serum in Asthma | Both and mouse model | Showed that endothelium-specific miR-1 regulates aeroallergen-induced mouse airway inflammation but clinical studies on miR-1 target genes did not show the expected correlations in all instances. | Tissue miR-1 levels had an inverse correlation with eosinophilia. | miRNA-mediated regulation of eosinophil trafficking |
| 31 | Gu et al/ 2020 (46) | Mice only | China | Sinonasal tissue of mice induced with CRS | Mouse model only | On dual luciferase assay, showed thatTPX2 is a target gene of miR-335-5p | miR-335-5p is poorly expressed in the nasal mucosal tissue of mice with CRS, and up-regulation of miR-335-5p can repress the activation of the AKT signaling pathway by targeting TPX2 | AKT signaling pathway |
| 32 | Du et al/ 2020 (50) | eCRSwNP-25; neCRSwNP-25; CRSsNP- 25; Controls- 25 and mouse model of CRS | China | NPs and ETM in CRSwNP; ETM in CRSsNP and ITM in controls | Both (in vitro with mouse model) | DEX treatment inhibited the expression of cytokines via inhibiting NF-kappaB/miR-155 | NF-kappaB/miR-155 was significantly elevated in the eCRSwNP group compared with all other groups, accompanied by the upregulation of cytokines: TNF alpha, IL-1, IL-4, IL-5 | NF-kappaB/miR-155 and response to glucocorticoids |
| 33 | Li et al/ 2019 (61) | CRSwNP-13; CRSsNP-12; Controls-11 | China | NPs from CRSwNP, MT from CRSsNP, ITM in controls | Both | Findings confirmed in vitro experiment using HNECs | expression levels of miR-21 and TGF-β1 mRNAs in CRSwNP were significantly higher than those in CRSsNP and controls. The miR-21 inhibitor, as well as the Akt-specific ­inhibitor, suppressed TGF-β1-induced EMT in HNECs | EMT and TGF beta-1 |
| 34 | Li et al/ 2019(59) | CRSwNP-5; CRSsNP-4; Controls-4 | China | NP tissue or ETM in CRS, ITM in controls | In vivo | Validation using RT-PCR (including validation of the miR‑4492 expression profile in CRS) | 6 differentially expressed miRNAs between CRSwNP and CRSsNP, one between CRSwNP and control and none between CRSsNP and controls. miR‑4492 is downregulated and IL‑10 is upregulated in NPs. | miR‑4492 is downregulated and IL‑10 is upregulated |
| 35 | Xuan et al/ 2019(52) | CRSwNP- 19; Controls-10 | China | Apex region of NP in CRSwNP and ITM in controls. | In vivo | No | 25 DE-miRNAs in CRSwNP (5 upregulated and 19 downregulated). Pathways affected by upregulated miRNAs included mucin type O- glycan biosynthesis and 5 of the downregulated miRNAs were involved in TGF-beta signaling pathway. | Mucin type O- glycan biosynthesis; TGF‑β1 signalling pathways identified |
| 36 | Liu et el/ 2019(34) | Gene Omnibus database  GSE36830; USA (CRSwNP-NP- 6; CRSwNP-UP- 6; CRSsNP-UP- 6; Controls- 6)  GSE72713 (eCRSwNP-3; neCRSwNP-3; Controls-3) | China | [GSE36830](https://www.ncbi.nlm.nih.gov/geo/query/acc.cgi?acc=GSE36830): NP for CRSwNP, UP for CRSsNP and controls  GSE72713: NPs in subjects and ‘normal sinus mucosa’ in controls | In vivo | lncRNA RP11-798M19.6 consistent in RT-qPCR on blood of CRSwNP | 265 differentially expressed lnc-RNAs and 994 DEmRNAs identified. lncRNA XLOC_010280 (regulates chemokine CCL18 and inﬂammation); RP11‑798M19.6 (regulates polypeptide N‑acetylgalactosaminyltransferase 7 / GALNT7 and cell proliferation) | Mucin type O-glycan synthesis, chemokine signalling pathway, staphylococcus aureus infection, cell adhesion molecules (CAMs), Ribosome, Butanoate metabolism |
| 37 | Ma et al/ 2018(41) | CRS- 37 (CRSsNP-12; Atopic CRSwNP- 12; Non-atopic CRSwNP-13); Controls-11 | China | Peripheral blood DC’s isolated and DC-naive CD4^+^ T cell co-cultures prepared | Both | Validated with a Dual-Luciferase Reporter Assay and western blot. | The expression of miR-150-5p and its target EGR2 (early growth response 2) was upregulated in DCs in CRS. | miR-150-5p upregulates EGR2 (transcription factor) |
| 38 | Liu et al/ 2018(43) | CRSwNP-20; Controls-20 | China | NP tissue from CRSwNP, ITM from controls. HNECs also cultured | Both | Validated using cell cultures | Transcription factor aryl hydrocarbon receptor (AHR), which is essential for modulating the immune response, was significantly higher in NPs and had an inverse correlation with the expression of miR124 miR124 may regulate body’s immune response in CRSwNP | miR124 downregulated and AHR (transcription factor, regulates enzymes like Cytochrome P450) upregulated |
| 39 | Yu et al/ 2018(42) | CRSwNP- 35 children; Controls-46 | China | NP from CRSwNP, ITM from controls. Also, serum and peripheral eosinophils in both groups. | In vivo | The dual luciferase reporter assay confirmed that TGF‑β1 was a target gene of miR-663. | TGF beta-1 mRNA and protein were significantly increased in all three types of specimens from pediatric patients with NPs and miR‑663 expression was significantly decreased in NP and peripheral blood eosinophils.miR‑663 may have regulatory effects on the pathogenesis of NP by regulating TGF‑β1 | miR‑663 decreased and its target TGF beta-1 increased (regulates cell proliferation, differentiation and growth, immune function, can modulate expression and activation of other growth factors including interferon gamma and tumor necrosis factor alpha) |
| 40 | Luo et al/ 2017(40) | CRSwNP-26 Controls-10 | China | Peripheral blood DCs | Both | Findings confirmed by in-vitro experiment | IL-10 expression of DCs was significantly lower, levels of miR-19a were higher in patients. Recombinant IL-4 suppressed the IL-10 expression in DCs, and this was abolished by blocking HDAC-11 or knocking down the miR-19a gene in DCs. miR-19a plays a role in suppression of IL-10 in peripheral DCs. | miR-19a suppresses IL-10 (pleiotropic effects in immunoregulation and inflammation. Down-regulates the expression of Th1 cytokines, enhances B cell survival, proliferation, and antibody production) |
| 41 | Ma et al/ 2015(39) | CRS-30; Controls-7  (CRS sub-divided into CRSsNP, atopic CRSwNP, non-atopic CRSwNP) | China | DCs form peripheral blood | In vivo | Verified by PCR | Different sets of DE-miRNAs in CRS types, when compared with Controls. 31 common DE-miRNAs among all three groups of CRS patients. 5 upregulated, 25 downregulated while MiR-1290 was down-regulated in CRSsNP but up-regulated in both atopic CRSwNP and non-atopic CRSwNP. | MiR-125b-5P, miR-15-5P, miR-210-3P, miR-708-5P, miR-126-3p |
| 42 | Xia et al/ 2015 (66) | CRSwNP-20; CRSsNP-20; Controls-5 | China | Unspecified for subjects; Controls from anterior ETM | In vivo | - | Differential expression of 7 mi-RNAs which were previously implicated in CRS were investigated. in all patients with CRS, miR-125b, miR-155 and miR-146a were up-regulated, while miR-92a, miR-26b and miR-181b were down-regulated. MiR124 expression levels were not found to have significant changes. | Some pathways identified: Renal cell carcinoma, mTOR signaling, Insulin signaling, ubiquitin mediated proteolysis, TGF beta signaling, PI3K-AKT signaling, MAPK signaling. |
| 43 | Cao et al/ 2015 (35) | Mouse model only | China | Mouse model and Human bronchial epithelial cell culture | In vitro | Bronchial epithelial cell culture showed siRNA targeting VEGF suppressed its mRNA and protein levels. | SiRNA targeting VEGF was delivered using chitosan based hydrogel, decreasing sinus mucosa thickness. | VEGF |
| 44 | Zhang et al/ 2012 (71) | eCRSwNP- 22; neCRSwNP- 22; CRSsNP- 30; Controls- 24 | China | NPs from CRSwNP; ETM from CRSsNP and ITM from controls | In vivo | - | Did not find any difference in mRNA and mi-RNA levels in CRS and controls, except PACT (Protein activator of the interferon-induced protein kinase) mRNA expression | - |
| 45 | Zhang et al/ 2012(54) | eCRSwNP-46; neCRSwNP-31; CRSsNP-43; Controls-50 | China | NP or ETM in CRS, ITM in controls. | Both | Confirmed that MiR-125b targets 4E-BP1 in ex-vivo airway epithelial cell culture | Distinct miRNA profiles between CRS and controls and between eCRSwNP and CRSsNP. Upregulated miR-125b may enhance type I IFN expression, potentially contributing to eosinophilia in eCRSwNP. | miR-125b enhances type I IFN expression (key part of the innate immune response with potent antiviral, antiproliferative and immunomodulatory properties. |

Abbreviations: circRNA: Circular RNA; ceRNAs: competing endogenous RNA; CRS: Chronic rhinosinusitis; CRSsNP: Chronic rhinosinusitis without nasal polyps; CRSwNP: Chronic rhinosinusitis with nasal polyps; DCs: Dendritic cells; DhMG: Differentially hydroxymethylated genes; DE-mRNA: Differentially expressed messenger RNA; DE-miRNA: Differentially expressed micro RNA; DE-lncRNA: Differentially expressed long non-coding RNA; DNMT: DNA Methyltransferase; ECM: Extra-cellular matrix; eCRSwNP: eosinophilic chronic rhinosinusitis with nasal polyps; EGF: Epidermal growth factor; EMT: Epithelial mesenchymal transition; ETM: Ethmoid mucosa; HNECs: Human nasal epithelial cells; ITM: inferior turbinate mucosa; miR: micro RNA; MSP: Methylation -specific Polymerase chain reaction; MT: Middle turbinate; neCRSwNP: non-eosinophilic chronic rhinosinusitis with nasal polyps; NLF; nasal lavage fluid; NP: nasal polyps; NPDFs: Nasal polyp dervived fibroblasts; PCR: Polymerase chain reaction; PM: Particulate matter; RT-PCR: Reverse-transcription polymerase chain reaction; UP: Uncinate process
